# De novo transcriptome analysis reveals insights into dynamic homeostasis regulation of somatic embryogenesis in upland cotton (*G. hirsutum* L.)

**DOI:** 10.1007/s11103-016-0511-6

**Published:** 2016-08-10

**Authors:** Wen-Han Cheng, Hua-Guo Zhu, Wen-Gang Tian, Shou-Hong Zhu, Xian-Peng Xiong, Yu-Qiang Sun, Qian-Hao Zhu, Jie Sun

**Affiliations:** 1 The Key Laboratory of Oasis Eco-Agriculture, College of Agriculture, Shihezi University, Shihezi, 832000 Xinjiang China; 2 Key Laboratory of Plant Secondary Metabolism and Regulation of Zhejiang Province, College of Life Sciences, Zhejiang Sci-Tech University, Hangzhou, 310018 Zhejiang China; 3 CSIRO Agriculture Flagship, GPO Box 1600, Canberra, 2601 Australia

**Keywords:** *Gossypium hirsutum* L., Transcriptome, Somatic embryogenesis (SE), Indole-3-butytric acid, Kinetin, Polyamines, Stress-response

## Abstract

Plant regeneration via somatic embryogenesis (SE) is the key step for genetic improvement of cotton (*Gossypium hirsutum* L.) through genetic engineering mediated by *Agrobacteria*, but the molecular mechanisms underlying SE in cotton is still unclear. Here, RNA-Sequencing was used to analyze the genes expressed during SE and their expression dynamics using RNAs isolated from non-embryogenic callus (NEC), embryogenic callus (EC) and somatic embryos (SEs). A total of 101, 670 unigenes were de novo assembled. The genes differentially expressed (DEGs) amongst NEC, EC and SEs were identified, annotated and classified. More DEGs were found between SEs and EC than between EC and NEC. A significant number of DEGs were related to hormone homeostasis, stress and ROS responses, and metabolism of polyamines. To confirm the expression dynamics of selected DEGs involved in various pathways, experiments were set up to investigate the effects of hormones (Indole-3-butytric acid, IBA; Kinetin, KT), polyamines, H_2_O_2_ and stresses on SE. Our results showed that exogenous application of IBA and KT positively regulated the development of EC and SEs, and that polyamines and H_2_O_2_ promoted the conversion of EC into SEs. Furthermore, we found that low and moderate stress is beneficial for proliferation of EC and SEs formation. Together, our global analysis of transcriptomic dynamics reveals that hormone homeostasis, polyamines, and stress response synergistically regulating SE in cotton.

**Electronic supplementary material:**

The online version of this article (doi:10.1007/s11103-016-0511-6) contains supplementary material, which is available to authorized users.

## Introduction

Crop losses due to multiple stresses have been predicted to be much greater than previously estimated in the coming future, especially due to climate change and industry development (Chen et al. 2015). Genetic improvement through breeding is the most efficient strategy for enhancing crop’s stress tolerance. In addition to traditional breeding approaches, genetic transformation provides an alternative method to improve pest and disease resistance, as well as yield, of crops (Markram et al. 1997; Wu et al. 2008). For the majority of crops, *Agrobacterium*-mediated transformation is the method of choice for genetic modification. The prerequisite of this method is to have a highly efficient and repeatable system for tissue culture and plant regeneration via somatic embryogenesis (SE) or organogenesis.

Many studies have investigated physiological and biochemical changes during SE in various plant species with a focus on understanding the mechanisms of gene regulation related to SE. These efforts identified genes differentially expressed in somatic embryos (SEs), highlighted the pathways likely to be involved in SE and discovered molecular or protein markers for SE (Mantiri et al. 2008). Some of the identified genes have been experimentally demonstrated to play an important role in SE. For instance, *Arabidopsis thaliana* plants transformed with the *SOMATIC EMBRYOGENESIS RECEPTOR-LIKE KINASE 1*(*AtSERK1*) gene showed a marked increase in SE compared to wild-type cultures (Hecht et al. 2001). In *Arabidopsis*, transcription factors *LEAFY COTYLEDON 1* (*LEC1*) is required for embryo development and *LEC2* induces somatic embryo development in vegetative cells. Ectopic expression of *LEC1* (Lotan et al. 1998) or *LEC2* (Stone et al. 2001) caused spontaneous formation of SEs on intact plants or explants. *AGL15* is another transcription factor that promotes SE in *Arabidopsis* (Harding et al. 2003). The *BABY BOOM* (*BBM*) gene can promote SEs in transgenic *Arabidopsis* and *Brassica* when ectopically overexpressed (Boutilier et al. 2002). *Medicago truncatula* SOMATIC EMBRYO RELATED FACTOR1 (*MtSERF1*) is essential for somatic embryo development and is induced by ethylene (Mantiri et al. 2008).

Cotton is one of the most important economic crops in the world, providing excellent natural fiber, and is also a source of oil. Cotton plantlets can be regenerated via SE using various combinations of plant growth-regulators, such as 2,4-Dichlorophenoxyacetic acid (2,4-D), indole-3-butyric acid (IBA), and naphthalene acetic acid (NAA) in combination with Kinetin (KT) (Sun et al. 2006). But cotton remains to be one of the notoriously recalcitrant plant species for plant regeneration via SE. So far, only few genotypes have been successfully used in gene transformation and genetic engineering. SE in cotton usually requires a very long culture time, and has a high frequency of abnormal embryos. To understand the molecular mechanisms underlying cotton SE and identify genes and critical pathways for cotton SE, suppression subtractive hybridization, macroarray and transcriptome during SE has been recently investigated (Xu et al. 2013; Yang et al. 2012; Zeng et al. 2006). Global transcriptome analyses suggested that auxin and cytokinin signaling pathways are critical in dedifferentiation of somatic cells and redifferentiation of SEs in cotton. In addition, stress-responsive genes and pathways were also found to be involved in SE (Yang et al. 2012). A more recent study has shown that the function of *GhCKI* (*CASEIN KINASE I*), a unique key regulatory factor strongly affecting cotton SE, is achieved by regulating auxin homeostasis through the network including other three genes, i.e. *GhLEC1, GhTCP15* (*TEOSINTE BRANCHED1-CYCLOIDEA-PCF15*) and *GhPIF4* (*PHYTOCHOME INTERACTING FACTOR4*) (Min et al. 2015). Despite these reports, we still know little about the molecular mechanisms underlying cotton SE.

In this study, we used Xinluzao 33 (*Gossypium hirsutum* L.), one of the main cotton cultivars planted in Xinjiang Province, China, to investigate the transcriptome profiles of tissues from different stages during SE. Plant regeneration via SE using Xinluzao 33 is problematic because of the long time required for embryogenic callus (EC) induction and the low ratio of somatic embryo differentiation. Our transcriptome studies not only confirmed the importance of auxin and cytokinin homeostasis in cotton SE, but revealed the importance of genes involved in the polyamine metabolic pathway and stress response in cotton SE. Our results provided foundation and clues for establishing a reproducible and highly efficient plant regeneration system that can be used in a diverse of cotton genotypes for fast and precise genetic improvement by genetic engineering.

## Materials and methods

### Plant materials and culture conditions

The method for cotton cultivar Xinluzao 33 SE used in this study has been previously described (Cheng et al. 2015; Sun et al. 2006). Non-embryogenic callus (NEC) (45 days), EC and SEs (a mixture of globular embryos, torpedo embryos and cotyledon embryos; SEs) (Supplementary Fig. 1) were sampled for RNA-Sequencing (RNA-seq), determinating the level of hormones, polyamines, H_2_O_2_, and qRT-PCR.

### RNA extraction, library construction, and RNA-Sequencing

The RNA library construction, and RNA-Sequencing was performed by Illumina Genome Analyzer IIx at Beijing Genomics Institute (BGI)-Shenzhen, China. The NEC, EC and SEs samples were biologically replicated once, i.e. in total six libraries were used in RNA-Seq.

### Analysis of sequencing data and identification of differentially expressed genes

The trimmed and filtered of raw reads and identification of differentially expressed genes in databases were based on a previous published paper (Xiang et al. 2010). Annotation of the differentially expressed unigenes was based on the *Gossypium raimondii* genome and *A. thaliala* genome (Kaul et al. 2000; Mayer et al. 1999; Wang et al. 2012). The genes mentioned in this study were also BLAST in other two recently reported *G. hirsutum* genome (TM-1) databases (Li et al. 2015; Zhang et al. 2015), in order to confirm the gene sequences and ID.

### Establishment of suspension cultures of uniform embryogenic callus

Suspension culture of uniform EC that was used in various physiological experiments outlined below was established following our previously described method (Cheng et al. 2015).

### Exogenous treatments of polyamines, H_2_O_2_, IBA, KT and simulated stresses

To determine the effects of polyamines, IBA (Indole-3-butytric acid) and KT on the conversion of EC into SEs, 60 µL of uniform embryogenic calli was cultured on somatic embryo induction medium supplemented with 1 mM of putrecine (Put), 1 mM H_2_O_2_, or 0.15 mg/L IBA + 0.15 mg/L KT. All reagents were purchased from Sigma Chemical Co. (St. Louis, MO). The simulated stress was performed with Phytagel (2.5, 4 g/L) or NaCl (0, 25, 50, 100 mM) in the MS medium. Each treatment was repeated three times biologically.

### Determination of polyamine concentration

Polyamine (PA) concentrations were determined using the high performance liquid chromatography (HPLC) method following the procedure previously described (Cheng et al. 2015). The assays were biologically repeated three times.

### Detection of hydrogen peroxide by 3, 3′-diaminobenzidine (DAB)

Detection of hydrogen peroxide by 3, 3′-diaminobenzidine (DAB) staining in each sample was achieved using our previously described method (Cheng et al. 2015). The assays were biologically repeated three times.

### Determination of IAA, KT, GA and ABA

IAA, KT, GA and ABA were determined using an ELISA Assay Kit and corresponding antibodies produced by the Jiancheng Biochemistry Company (Nanjing, China) according to the manufacturer’s instructions. The assays were biologically repeated three times.

### RNA extraction and quantitative real time PCR (qRT-PCR)

RNA extraction, qRT-PCR and expression assay methods were described in our previous paper (Cheng et al. 2015). The means of the three biological experiments were calculated as the expression level of the genes analyzed. Primers used in qRT-PCR are listed in Supplementary Table 1.

### Statistical analysis

Analysis of variance was performed using the SPSS16.0 statistical analysis package. Differences between means were compared by Fisher’s least-significant-difference test at the 5 and 1 % probability levels.

## Results

### RNA-sequencing and transcriptome de novo assembly

Generally, the process of cotton SE includes NEC induction, EC induction, differentiation of SEs, and plant regeneration (Supplementary Fig. 1). To investigate the transcriptome dynamics during cotton SE, we performed RNA-Seq using mRNA extracted from NEC, EC and SEs.

In total, 168,196,388 75-bp paired-end raw reads were generated. For all three samples, the percentage of nucleotides with a quality score above 20 (Q20) was over 98.00 %, the percentage of reads containing N was lower than 0.02 % and the GC percentage was ~44 % (Supplementary Table 2). After filtering, 160,407,732 clean reads were retained and used in transcriptome de novo assembly using the Trinity program. As a result, 97,857, 87,997 and 84,176 unigenes were assembled in NEC, EC and SEs, respectively (Supplementary Table 3). Combining unigenes from all three samples, in total, 101,669 non-redundant unigenes were assembled. The majority of unigenes were 200–500 bp in length, and the numbers of unigenes and contigs decreased with the increase of the unigene length (Supplementary Table 3).

### Functional annotation of unigenes

**Fig. 1:**
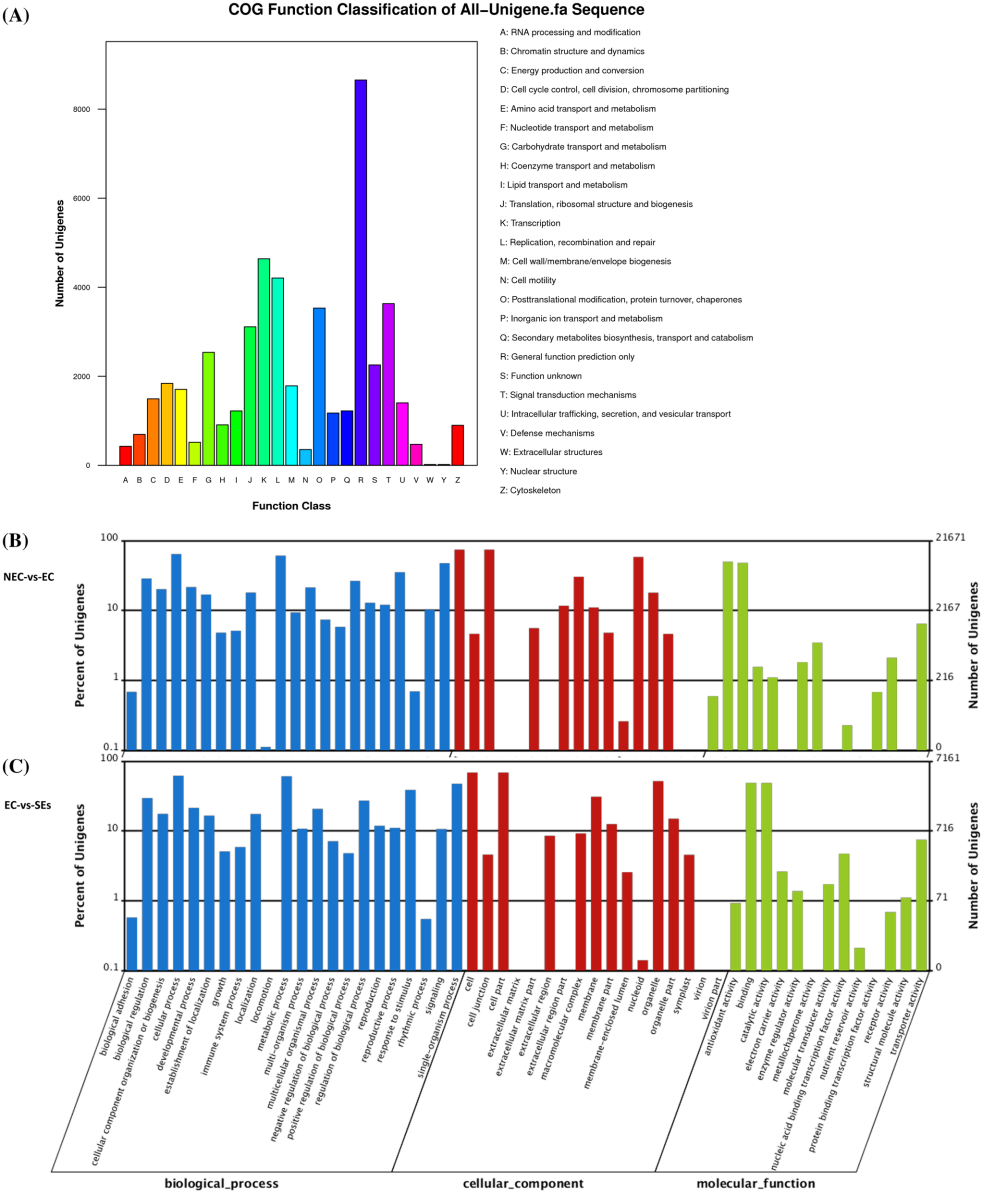
Function classification of all unigenes in COG and GO. **a** COG function classification. **b** GO classification of unigenes in embryogenic callus compared with non-embryogenic callus at three levels. **c** GO classification of unigenes in somatic embryos compared with embryogenic callus at three levels

Unigenes were functionally annotated by searching against the NT, Swiss-Prot, KEGG, COG and GO databases using the Blast2GO algorithm with an E value ≤10^−5^. Of the total 101,669 non-redundant unigenes, 61,389, 43,333, 39,721, 25,923 and 50,884 had a hit in the NR, NT, Swiss-Prot, KEGG, COG and GO database, respectively. The predicted amino acid sequences of the unigenes were further aligned against the Pfam database (E value ≤10^−6^) using the HMMER software. Altogether, 74,306 (73 %) unigenes had an annotated function. According to the COG (http://www.ncbi.nlm.nih.gov/COG) functional classification, the unigenes assembled were categorized into 25 groups, and were mostly covered by the inherent function, such as RNA processing, transcription, replication, recombination, repair and nucleotide or amino acid metabolism (Fig. 1a). Some unigenes were annotated to be involved in signal transduction, cell cycle control and cell division. Genes related to plant growth, developmental progress and responses to stimuli were also well covered by the de novo assembly (Fig. 1a).

Based on GO (http://www.geneontology.org) classification, the majority of the unigenes under cellular component were involved in cell, cell part, organelle, membrane, organelle part, macromolecular complex, membrane part, cell junction and extracellular region. Molecular function categories were mostly clustered in binding, catalytic activity and transporter activity. The annotated unigenes clustered under biological process mainly involved in cellular process, metabolic process, single-organism process, response to stimulus and regulation of biological process. Comparing EC with SEs, there were more unigenes involved in locomotion at biological process level, more unigenes involved in macromolecular complex and nucleoid at cellular component level, and more unigenes involved in structural molecule activity at molecular function level (Fig. 1b, c).

### Comparative analysis of differentially expressed unigenes in NEC, EC and SEs

**Fig. 2:**
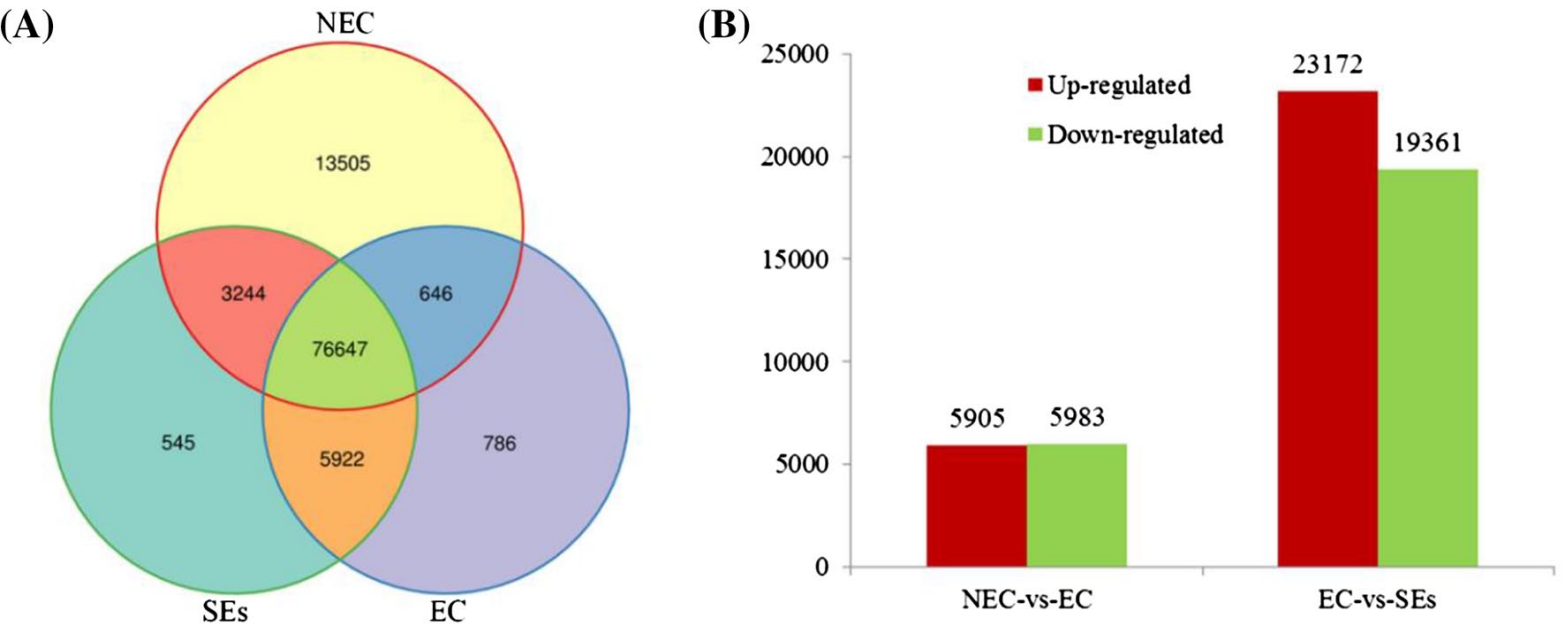
Statistical analysis of differentially expressed unigenes in NEC, EC and SEs. **a** The *venn diagram* of the unigenes in NEC, EC and SEs. **b** Statistical analysis of up/down regulated unigenes in NEC vs EC and EC vs SEs

Growth of NEC is in the process of dedifferentiation of vegetative growth, while formation of EC and development of SEs are regarded as in the process of dedifferentiation of reproductive growth. Spatial analysis was also performed on differentially expressed unigenes to ascertain the degree of overlap existing between the three different developmental processes during cotton SE. There were 94,020, 84,001 and 86,358 differentially expressed unigenes in NEC, EC and SEs (Fig. 2a). Among these, more than half (75.7 %) of the differentially expressed genes were present in all three developmental processes. Significant numbers of genes were present in one developmental process only: 13,505 unigenes were only differentially expressed in NEC, 786 differentially expressed unigenes were only switched on/off EC, and 545 genes changed their expression level in SEs, which suggested that distinct spatial transcriptional profiles were present (Fig. 2a). Compared with NEC, EC had 5905 and 5983 unigenes down- and up-regulated, respectively. Compared with EC, SEs had 23,172 down-regulated unigenes and 19,361 up-regulated unigenes (Fig. 2b). These results suggest that the cotton transcriptome undergoes significantly dynamic changes during SE, particularly during the period from EC to SEs.

### Pathways of differentially expressed unigenes based on KEGG

In total, there were 101,669 differentially expressed unigenes amongst NEC, EC and SEs, which were mainly involved in 127 pathways based on KEGG annotation. The pathways related to metabolites (e.g.purine, glycerophospholipid, starch and sucrose, ether lipid, amino sugar, nucleotide sugar, pyruvate) and biosynthesis of secondary metabolites (e.g. phenylpropanoid, stilbenoid, diarylheptanoid, gingerol and flavonoid), covered almost half of the differentially expressed unigenes. A considerable number of unigenes involved in DNA repair and replication, transcription, mRNA translation and protein folding, sorting and degradation were differentially regulated during the development of EC and SEs. Additionally, some stimulus pathways (e.g. plant hormone signal transduction and plant-pathogen interaction), ubiquitin mediated proteolysis, oxidative phosphorylation, ABC transporters and circadian rhythm were also found to be covered by differentially expressed unigenes. The top 30 pathways with the most number of differentially expressed unigenes are shown in Table 1.

**Table 1:** Pathways of differentially expressed unigenes annotated in KEGG

No.	Pathway annotated in KEGG	Differentially expressed unigenes with pathway annotation (101,669)	P value	Q value	Pathway ID	Level 1	Level 2
1	Metabolic pathways	3772 (20.81 %)	0.000294816	1.70E−03	ko01100	Metabolism	Global map
2	Biosynthesis of secondary metabolites	1858 (10.25 %)	7.63E−08	1.21E−06	ko01110	Metabolism	Global map
3	Plant hormone signal transduction	1098 (6.06 %)	0.002668437	1.21E−02	ko04075	Environmental information processing	Signal transduction
4	Plant-pathogen interaction	1090 (6.01 %)	0.01333107	5.29E−02	ko04626	Organismal systems	Environmental adaptation
5	RNA transport	1063 (5.86 %)	0.02651119	8.63E−02	ko03013	Genetic information processing	Translation
6	Spliceosome	735 (4.05 %)	0.05205961	1.54E−01	ko03040	Genetic information processing	Transcription
7	Ribosome biogenesis in eukaryotes	655 (3.61 %)	0.949479	1.00E+00	ko03008	Genetic information processing	Translation
8	RNA degradation	618 (3.41 %)	0.9999995	1.00E+00	ko03018	Genetic information processing	Folding, sorting and degradation
9	Protein processing in endoplasmic reticulum	519 (2.86 %)	0.999999	1.00E+00	ko04141	Genetic information processing	Folding, sorting and degradation
10	Purine metabolism	457 (2.52 %)	0.3051416	5.96E−01	ko00230	Metabolism	Nucleotide metabolism
11	Glycerophospholipid metabolism	422 (2.33 %)	0.5176856	8.32E−01	ko00564	Metabolism	Lipid metabolism
12	Pyrimidine metabolism	418 (2.31 %)	0.6995854	1.00E+00	ko00240	Metabolism	Nucleotide metabolism
13	Endocytosis	405 (2.23 %)	0.9955326	1.00E+00	ko04144	Cellular Processes	Transport and catabolism
14	Ubiquitin mediated proteolysis	347 (1.91 %)	0.9976985	1.00E+00	ko04120	Genetic information processing	Folding, sorting and degradation
15	Ribosome	344 (1.9 %)	1	1.00E+00	ko03010	Genetic information processing	Translation
16	Starch and sucrose metabolism	333 (1.84 %)	0.1683237	3.96E−01	ko00500	Metabolism	Carbohydrate metabolism
17	Ether lipid metabolism	256 (1.41 %)	0.1162658	3.08E−01	ko00565	Metabolism	Lipid metabolism
18	RNA polymerase	237 (1.31 %)	0.8641065	1.00E + 00	ko03020	Genetic information processing	Transcription
19	Oxidative phosphorylation	228 (1.26 %)	0.8898479	1.00E+00	ko00190	Metabolism	Energy metabolism
20	Phagosome	219 (1.21 %)	0.6801155	1.00E+00	ko04145	Cellular processes	Transport and catabolism
21	Phenylpropanoid biosynthesis	235 (1.3 %)	2.96E−09	7.62E−08	ko00940	Metabolism	Biosynthesis of other secondary metabolites
22	Glycolysis/gluconeogenesis	233 (1.29 %)	2.46E−05	1.74E−04	ko00010	Metabolism	Carbohydrate metabolism
23	Homologous recombination	199 (1.1 %)	0.000677679	3.59E−03	ko03440	Genetic information processing	Replication and repair
24	Amino sugar and nucleotide sugar metabolism	196 (1.08 %)	0.8993965	1.00E+00	ko00520	Metabolism	Carbohydrate metabolism
25	ABC transporters	186 (1.03 %)	0.842251	1.00E+00	ko02010	Environmental information processing	Membrane transport
26	Circadian rhythm-plant	185 (1.02 %)	0.8448579	1.00E+00	ko04712	Organismal systems	Environmental adaptation
27	Pyruvate metabolism	184 (1.01 %)	0.4801359	8.11E−01	ko00620	Metabolism	Carbohydrate metabolism
28	Flavonoid biosynthesis	176 (0.97 %)	1.46E−05	1.32E−04	ko00941	Metabolism	Biosynthesis of other secondary metabolites
29	Arginine and proline metabolism	140 (0.77 %)	0.002212563	1.04E−02	ko00330	Metabolism	Amino acid metabolism
30	Fatty acid biosynthesis	67 (0.37 %)	0.3003386	5.96E−01	ko00061	Metabolism	Lipid metabolism

### Transcription factors (TFs) involved in cotton SE

**Fig. 3:**
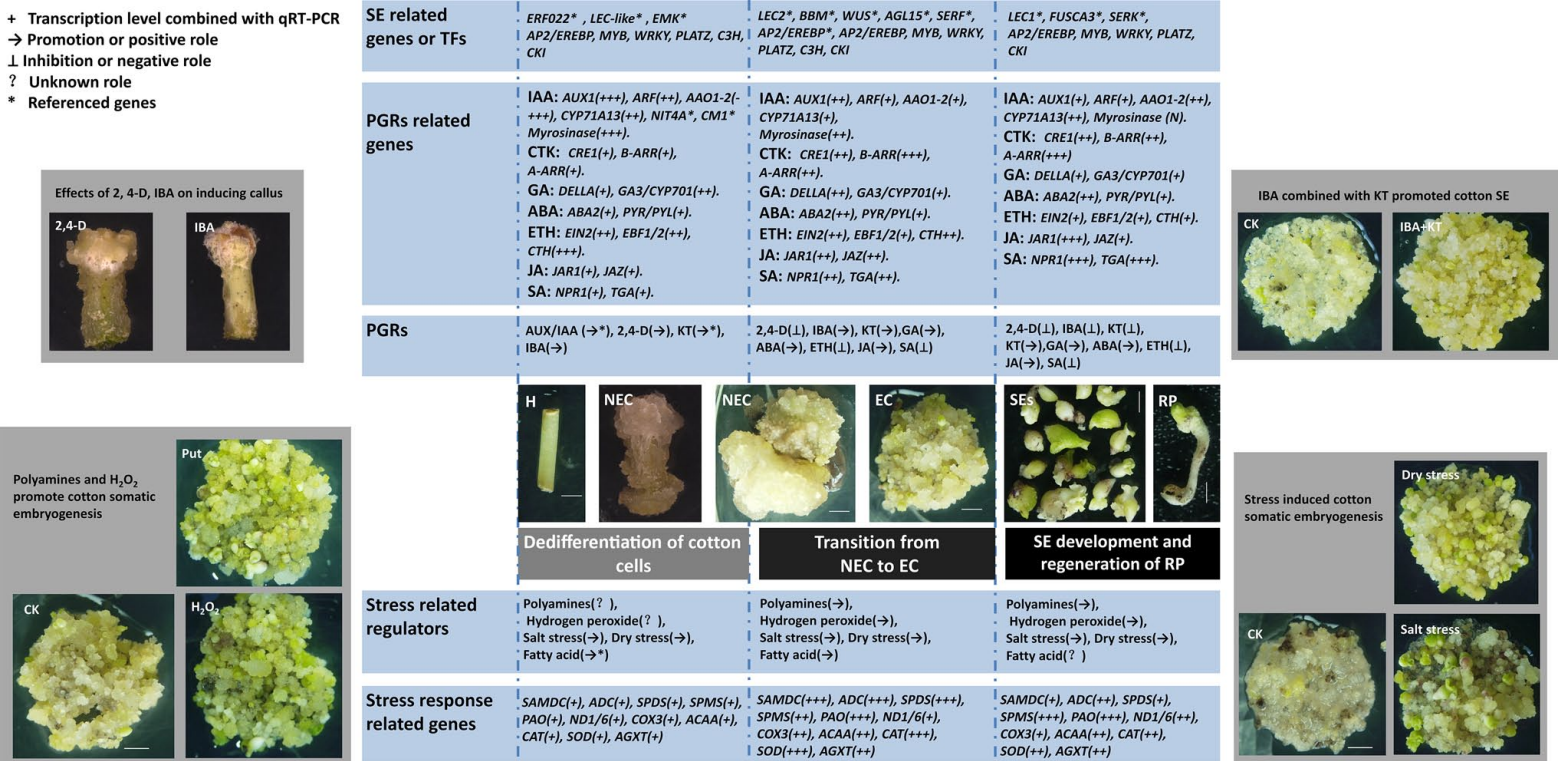
Differentially expressed TFs, PGRs, stress response related genes during cotton SE and the effects of PGRs, polyamines, H_2_O_2_ and stresses on cotton SE

We identified 302 and 112 differentially expressed TFs in EC (EC vs NEC) and SEs (SEs vs EC), respectively. Among these TFs, members of the following families were overrepresented: AP2/EREBP, C3H/C2H2/C2C2-Dof zinc finger proteins, MYB domain-containing proteins, and WRKY domain transcription factors (Fig. 3 and Supplementary Table 4). In addition, some TFs, such as *SERK, LEC, WUS, BBM, CKI* and *AGL15*, have been previously demonstrated to play a role in SE were also found to be differentially expressed during cotton SE (Fig. 3; Table 2). These TFs are multifunctional regulators in both zygotic and SE with a role in hormone signaling and stress responses (Mahdavi-Darvari et al. 2015). Some of these TFs have been used as markers of totipotency in plant species. These results suggest that dynamic changes of the expression levels of plant transcription factors are critical for differential and development of EC and SEs.

**Table 2:** Differentially expressed unigenes showing SE-specific expression

Gene ID	Gene name	Annotation in KEGG	Log2 fold change	
			NEC vs EC	SEs vs EC
CL11109.Contig1_All	*ADC*	Polyamines	1.2	−1
CL1916.Contig4_All	*SPDS*	Polyamines	3.6	−2.3
CL1916.Contig5_All	*SPMS*	Polyamines	4.2	4.9
Unigene22850_All	*SAMDC*	Polyamines	11.6	−10.6
Unigene16582_All	*PAO*	Polyamines	2.6	1
CL2187.Contig18_All	*AUX1*	AUXIN signal	−10.6	−4.7
CL5808.Contig3_All	*AUX*/*IAA*	AUXIN signal	−13	1.2
Unigene18856_All	*ARF*	AUXIN signal	−12.3	11.3
CL4407.Contig1_All	*TRIT1*	Cytokinin synthesis	2.1	−1.5
Unigene2420_All	*CRE1*	Cytokinin signal	14.0	2.9
Unigene17496_All	*B-ARR*	Cytokinin signal	15.4	14.7
Unigene11345_All	*A-ARR*	Cytokinin signal	10.6	11.4
Unigene19592_All	*DELLA*	Gibberellin signal	12.9	−1.4
Unigene27460_All	*GA3*/*CYP701*	Gibberellin synthesis	−2.0	3.9
CL7589.Contig3_All	*ABA2*	ABA synthesis	11.3	2.1
CL5280.Contig2_All	*PYR*/*PYL*	ABA signal	6.6	−4.7
Unigene217_All	*EIN2*	Ethylene signal	−1.3	−1
Unigene2682_All	*EBF1*/*2*	Ethylene signal	−5.6	−5
Unigene38355_All	*CTH*	Ethylene synthesis	−11.9	–
Unigene6360_All	*JAR1*	Jasmonic acid signal	1.8	1.2
Unigene28086_All	*JAZ*	Jasmonic acid signal	5.7	−4.6
Unigene32397_All	*NPR1*	Salicylic acid signal	−13.1	−2.3
CL3069.Contig6_All	*TGA*	Salicylic acid signal	−6.1	−5
CL11268.Contig2_All	*ND1*	Oxidative phosphorylation	3	1
CL4559.Contig2_All	*ND6*	Oxidative phosphorylation	8.2	−3.7
CL3850.Contig1_All	*COX3*	Oxidative phosphorylation	14.4	−12.9
CL5613.Contig1_All	*ACOX*	Stress-response	1.8	2.7
CL2833.Contig1_All	*CAT*	Stress-response	3.5	−2.9
CL5637.Contig1_All	*APX*	Stress-response	5.1	4.3
CL8467.Contig1_All	*SOD*	Stress-response	1.8	−1.1
CL7474.Contig1_All	*CDPK*	Stress-response	2.9	−3.2
CL10919.Contig1_All	*HSP90*	Stress-response	6.8	−1.3
Unigene15666_All	*SGT1*	Stress-response	2.2	2.3
CL3327.Contig4_All	*FabH*	Fatty acid biosynthesis	2.7	−1.9
Unigene24187_All	*accC*	Fatty acid biosynthesis	4.8	4.5
Unigene8973_All	*FatB*	Fatty acid biosynthesis	3.1	−2.5
Unigene26934_All	*FabD*	Fatty acid biosynthesis	3.8	2.6
Unigene9530_All	*SERK*	Transcription factor	1.8	2.4
CL11093.Contig1_All	*LEC1*	Transcription factor	1.7	3.1
Unigene10107_All	*WUS*	Transcription factor	4.1	4.1
Unigene26347_All	*BBM*	Transcription factor	2.9	1.8
CL5242.Contig1_All	*AGL15*	Transcription factor	1.2	1.1
CL7829.Contig6_All	*CKI*	Transcription factor	2.8	−1.9

For homologous genes, the unigene with the highest absolute value of the fold change was selected. The positive and negative number represent up- and down-regulation, respectively. – Indicates unchanged

### Auxin and cytokinin played an important role in the transition from NEC to EC and from EC to SEs

A number of genes involved in auxin and cytokinin synthesis and signal transduction pathways were differentially expressed from NEC to EC and from EC to SEs (Fig. 3; Table 2). For example, from NEC to SEs, the expression levels of *AAO1-2* and *CYP71A13*, genes involved in IAA biosynthesis, were first down-regulated in EC and then slightly up-regulated in SEs. The gene encoding myrosinase, an IAA biosynthesis related enzyme, was continuously down-regulated from NEC to SEs (Fig. 3). For *AUX1, AUX*/*IAA* and *ARF*, genes involved in auxin signal transduction, the highest expression levels were observed in NEC. From EC to SEs, the expression level of *AUX1* was further decreased whereas the expression level of *ARF* was increased (Table 2; Supplementary Fig. 2). *TRIT1*, a gene related to cytokinin (zeatin) biosynthesis, was up-regulated in EC but down-regulated in SEs. *CRE1* involved in cytokinin signal transduction was up-regulated in EC and remained high in SEs (Fig. 3; Table 2; Supplementary Fig. 2). Another two genes involved in cytokinin signal transduction, *A-ARR* and *B-ARR*, showed a similar expression pattern as *CRE1*, although compared to EC, SEs had a decreased level of *A-ARR* (Supplementary Fig. 2).

**Fig. 4:**
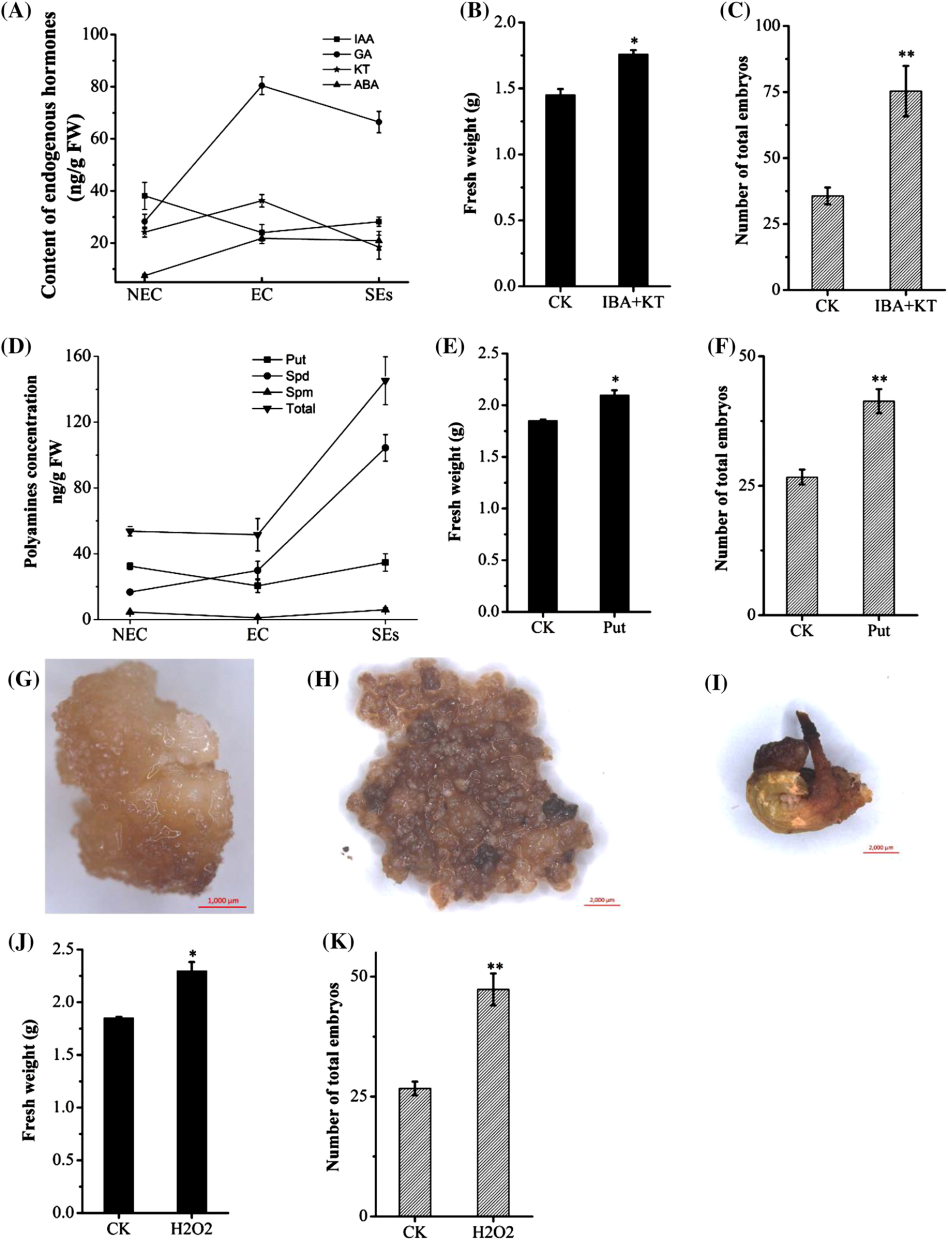
Comparison of endogenous IAA, KT, GA, ABA, polyamines and H_2_O_2_ at different stages of SE and their effects on the growth of embryogenic callus and somatic embryos. **a** Determination of the endogenous level of IAA, KT, GA and ABA with ELLISA assay. **b** Effect of IBA and KT on the growth of somatic embryogenic callus. **c** Effect of IBA and KT on the number of somatic embryos. **d** Determination of polyamines using the HPLC assay. **e** Effect of put on the growth of somatic embryogenic callus. **f** Effect of put on the number of somatic embryos. **g** DAB staining of H_2_O_2_ in non-embryogenic callus. **h** DAB staining of H_2_O_2_ in embryogenic callus. **i** DAB staining of H_2_O_2_ in somatic embryo. **j** Effect of H_2_O_2_ on the growth of somatic embryogenic callus. **k** Effect of H_2_O_2_ on the number of somatic embryos. * and ** indicate significant differences compared with the control (CK) at P < 0.05 and P < 0.01, respectively, according to the LSD multiple range test

To monitor changes of auxin and cytokinin during SE, we measured the concentrations of endogenous IAA and KT in NEC, EC and SEs by ELLISA assay. The IAA concentration was the highest in NEC and decreased in EC and then slightly increased in SEs. The concentration of KT increased in EC and then significantly decreased in SEs. These results were consistent with the expression levels of *AAO1-2, CYP71A13* and *TRIT1* in these samples (Figs. 3, 4a). SE can be controlled using different combinations of 2,4-D and KT, or IBA and KT (Fig. 3). With the reduction of 2,4-D concentration in the media, the callus became loose and granular in texture and yellow-green in color, a sign for efficient EC induction. The combination of IBA and KT significantly increased the fresh weight and the number of total embryos (Fig. 4b, c). Together, these results confirmed that auxin and cytokinin are critical regulators during cotton SE and that the role of IBA and KT in transition from NEC to SEs was supported by their dynamic changes of expression levels during the transition.

### Polyamines promoted cotton SE

Based on KEGG analysis and GO annotation, the genes involved in polyamine synthesis or metabolism, including *SAMDC, ADC, SPDS, SPMS* and *PAO*, were found to be differentially expressed during cotton SE. All of these genes, particularly *SAMDC* and *PAO*, showed a higher expression level in EC compared with NEC. The expression levels of *SAMDC, ADC* and *SPDS* were decreased in SEs compared with EC, but the expression level of *SPMS* was further increased in SEs while that of *PAO* remained unchanged in SEs (Fig. 3; Table 2 and Supplementary Fig. 2).

We measured the concentrations of the three common PAs (Put, Spd and Spm) in NEC, EC and SEs. The concentrations of Spd and total PA showed significant increase in EC and SEs compared to NEC. Although the levels of all three types of PAs and the total PAs did not show significant difference between NEC and EC, the levels of Spd as well as the total PAs were significantly increased in SEs whereas the levels of Put and Spm largely remained unchanged in SEs (Fig. 4d). Nevertheless, application of exogenous Put in the medium significantly increased the growth of EC and the number of SEs (Fig. 4e, f). These results demonstrated that polyamines played a critical role in promoting the conversion of EC to SEs, Put and Spd might function similarly and are mutually substitutable.

### Cotton SE is regulated by reactive oxygen species (ROS)

According to our transcriptomic data, some oxidative phosphorylation related genes, including NADH dehydrogenase, succinate dehydrogenase, cytochrome C oxidase and A/F/V-type ATPase, were differentially expressed amongst NEC, EC and SEs (Fig. 3; Table 2). The NADH dehydrogenase related genes, such as the *ND, Ndufs* and *Ndh* family genes were up-regulated in EC and SEs compared to NEC. The genes in succinate dehydrogenase pathway, such as *SDHC, SDHA* and *SDHB* were also differentially expressed during cotton SE. The cytochrome C oxidase encoding genes, including *COXs, ISP, Cyt1*/*b, COR1* and *QCRs* showed an up-regulated pattern in EC and SEs. The ATPase (includes A, F and V-type) genes, such as *beta, alpha, delta* and *OSCP* had a relatively high expression level in EC (Fig. 3; Table 2 and Supplementary Fig. 2). Differential expression of genes related to oxidative phosphorylation indicated that energy metabolism is very active in the transition from NEC to EC and development of SEs.

Additionally, genes encoding the protective system enzymes, including CAT, SOD, POD and APX were up-regulated in EC and SEs (Fig. 3; Table 2 and Supplementary Fig. 2). These genes are involved in the antioxidant system and might play a role in maintaining ROS homeostasis during the differential and development of EC and SEs. The concentration of endogenous H_2_O_2_ was slightly higher in EC and SEs than in NEC as revealed by DAB staining (Fig. 4). Applying 1 mM H_2_O_2_ in the medium significantly increased the number of SEs (Fig. 4j, k), suggesting that ROS played a crucial role in the development of EC and SEs during cotton SE.

### Stress induced cotton SE

We found that many stress response related genes, such as *CDPK, HSP90, SGT* and genes encoding the protective system enzymes like NOX, SOD, CAT, POD, APX, SAMDC, PAO were differentially expressed during cotton SE (Fig. 3; Table 2). To know the role of stresses in differential and development of SEs, the effects of different concentrations of NaCl (0–100 mM) and simulated drought stress on cotton SE were investigated by adding Phytagel (4 g/L) into the MS medium. After 4-week of NaCl treatment, EC growth was promoted by relatively lower concentrations of NaCl (≤50mM) but inhibited by the high concentration of NaCl (100 mM). Compared to the control, the 25 and the 50 mM treatments slightly and significantly increased the fresh weight of EC, respectively, but the 100 mM treatment had very significant adverse effect on EC growth (Supplementary Fig. 3), and no EC finally survived in the MS medium containing 100 mM of NaCl. Consequently, after 4-weeks of culture, significantly more SEs were observed in the medium containing 50 mM of NaCl than in the control medium (Fig. 3). Similarly, in the drought stress treatment, more SEs in the medium with 4 g/L Phytagel were observed than in the control medium (2.5 g/L) after 4-weeks of culture (Fig. 3 and Supplementary Fig. 4). These results suggest that low and moderate stresses were beneficial for EC proliferation and differential of SEs.

### Fatty acid biosynthesis and metabolism during cotton SE

Fatty acids, as a type of protective chemical in reproduction and seed preservation, play a very significant role in the evolution and developmental processes. Fatty acids in plants, as in all other organisms, are the major structural components of membrane phospholipids and triacylglycerol storage oils. During cotton SE, genes related to fatty acid biosynthesis, including *FabD, FabH*, a*ccC* and *FatB*, were up-regulated in EC compared to NEC, but down-regulated in SEs compared to EC (Table 2 and Supplementary Fig. 2). The genes involved in metabolism of fatty acids, such as *ACOX1, ACOX3, fadA, fadB* and *fadI* showed an expression pattern opposite to those in EC and SEs. These results indicated that fatty acids accumulated during the development of EC, a situation similar to initialization of plant seeds.

## Discussion

### Transcription factors regulate SE

During the past three decades, numerous transcription factors and protein kinases, and a wide range of hormones have been shown to play a role in SE in different plant species (El Ouakfaoui et al. 2010; Mahdavi-Darvari et al. 2015). The *SERK* family genes, *LEC, WUS* and genes encoding germin-like proteins were implicated in the signal transduction pathway and transcriptional regulation during SE (aan den Toorn et al. 2015; Gaj et al. 2005; Podio et al. 2014; Zuo et al. 2002). *LEC1, LEC2* and *FUS3* are key genes that control SE process (Fambrini et al. 2006; Gaj et al. 2005). The capacity of SE is completely repressed in double (*lec1 lec2, lec1 fus3, lec2 fus3*) or triple (*lec1 lec2 fus3*) mutants in *Arabidopsis thaliana* (Gaj et al. 2005). The expression level of *LEC2*/*FUS* changes rapidly in response to auxin treatment (Stone et al. 2008), suggesting that *LEC*/*FUS* may be the upstream genes in the auxin signaling pathway (Gaj et al. 2005; Stone et al. 2001). *BBM* was similar to the *AP2*/*ERF* family genes and expressed preferentially in developing embryos (Boutilier et al. 2002). Over-expression of *AGL15* enhanced production of secondary embryos from cultured zygotic embryos in *A. thaliana* (Harding et al. 2003). In our study, these referenced TF genes were found to be differentially expressed during cotton SE (Fig. 3; Table 2).

We found that 302 and 112 TFs were differentially expressed during the transition from NEC to EC and the development of SEs, respectively. According to Yang et al. (2012), of the total 466 differentially expressed TFs during cotton SE, 338 were found during the transition from NEC to EC and 342 were found during development of SEs. More differentially expressed TFs identified by Yang et al. (2012) during development of SEs could be due to the difference in the samples used. Yang et al. (2012) used globular embryos, torpedo embryos and cotyledon embryos individually, while we used a mixture of three types of embryos, which could have compromised the sensitivity of DEG identification.

### Auxin and cytokinin are important regulators for cotton SE

Auxin and cytokinin are critical plant growth regulators (PGRs) for the induction of SE. Their regulatory role is probably achieved by establishing auxin and cytokinin gradients during the induction phase of SE, and is essential for initiating dedifferentiation and cell division of already differentiated cells before they can express embryogenic competence (Sharma et al. 2008; Su et al. 2011). Despite the absolute requirement for exogenous auxin to sustain growth of plant cells cultured in vitro, cultured plant cells produce substantial amounts of the native auxin, IAA. Comprehensive studies have been conducted on callogenesis from several cotton cultivars using various explants, and different combinations of growth regulators. The concentrations of IAA and KT were different in NEC, EC and SEs during cotton SE. Concentration of IAA decreased but concentration of KT increased in the EC stage compared to the NEC stage, consistent with the expression levels of genes involved in IAA and cytokinin biosynthesis (Table 2). This dynamics of auxin and cytokinin played a very important role in the transition from NEC to EC and the development of SEs (Yang et al. 2012; Xu et al. 2013). Previous studies had also shown that sharp changes in endogenous auxin and cytokinin levels might be one of the first steps leading to SE (Thomas et al. 2002). Our experimental results on the role of exogenously applied IBA and cytokinin on SE (Fig. 3) were consistent with previous studies, i.e. induction of NEC from hypocotyl requires addition of 2,4-D into the medium, but to induce EC and SEs, the concentration of 2,4-D in the medium was decreased to a lower level or replaced by combination of IBA and KT (Kumar et al. 2015; Sun et al. 2006). However, the role of auxin and cytokinin is not achieved alone, but by interacting with other pathways and components involved in differential and development of SEs, such as their upstream TF regulators, hormone signaling pathways and ROS. Further studies are required to understand the interactive relationship of these components involved in the complex network regulating cotton SE.

### Relationship between polyamines and SE

Polyamines have been previously linked to plant stress response and SE (Yoda et al. 2009). Many studies have demonstrated the importance of PAs for SE in several plant species (De-la-Peña et al. 2015; Feirer et al. 1984), probably by promoting cellular differentiation during SE (Gemperlová et al. 2009; Montague et al. 1978, 1979; Niemenak et al. 2012). The concentrations of polyamines increased during the early stages of SE in conifers but decreased during the late stages (Gemperlová et al. 2009; Paul et al. 2009). Application of putrescine, spermidine and spermine significantly improved SE in *Theobroma cacao* L, *Citrus sinensis* and *Hurst Ecotype* (Malá et al. 2012; Silva et al. 2009; Wu et al. 2009). In our study, the levels of Spd significantly increased in SEs and the levels of Put and Spm almost did not change, a result reflected by the expression levels of the genes, such as *SAMDC, ADC* and *SPDS*, encoding enzymes catalyzing the production of PAs (Table 2; Fig. 3 and Supplementary Fig. 2). Both our transcriptomic and qRT-PCR data showed that the key genes for biosynthesis of PAs were expressed at a higher level in EC (e.g. *ADC* and *SAMDC*), or in both EC and SEs (e.g. *SPDS* and *SPMS*; Table 2; Fig. 3). Both ADC mRNA and protein were localized in dividing cells of embryo meristems, probably required for mitosis (Vuosku et al. 2006). Exogenous application of Put and Spm enhanced the growth of embryogenic cultures of *Araucaria angustifolia* and significantly affected the endogenous concentrations of PAs, IAA and ABA in embryogenic tissues (Steiner et al. 2007). Our results also showed that exogenous application of Put enhanced the growth and the development of cotton SEs (Fig. 3), which could be achieved by PAs-mediated regulation of specific physiological processes important for cellular differentiation.

### Possible roles of other hormones in cotton SE

In addition to auxin and cytokinin, genes related to biosynthesis and signaling of other hormones, including gibberellin, abscisic acid, ethylene, brassinosteroid, jasmonic acid and salicylic acid, were also differentially expressed in NEC, EC and SEs (Table 2 and Supplementary Fig. 5). The gibberellin synthesis and signal transduction genes, *GA3*/*CYP701* and *KAO*, were down-regulated in EC but up-regulated in SEs. Endogenous GA was at higher levels in EC and SEs than NEC (Fig. 4a). *DELLA*, a negative regulatory factor (Ueguchi-Tanaka et al. 2008), was up-regulated in EC and down-regulated in SEs. ABA synthesis and signal transduction related genes were up-regulated in EC and SEs, including *ABA2, NCED, PP2C, ABF* and *PYR*/*PYL*. ABA levels increased in EC and SEs, which was consistent with the expression levels of the genes for ABA synthesis (Fig. 4a). SE of carrot (*Daucus carota* L.) and *Hevea brasiliensis* required ABA as the source of growth regulator in medium (Dunstan et al. 1988; Etienne et al. 1993). Ethylene and polyamines shared the common precursor SAM, always played a competitive role in SE (Bai et al. 2013; Linkies and Leubner-Metzger 2012). Genes related to ethylene synthesis and signal transduction, *CTH, cysK, GOT1, EIN2* and *EIN3*, were down-regulated in EC and SEs compared with NEC, and our data showed a competitive role between PAs and ethylene in cotton SE. JA synthesis and signal transduction related genes, such as *JAR1, COI-1* and *JAZ* were up-regulated in SEs, but these genes were down-regulated in EC. SA related genes had an opposite expression pattern with JA in such process. Genes of *CYP90C1*/*ROT3, CYP85A1*/*BR6OX1* and *CYP734A1*/*BAS1*, involved in BRs synthesis and signaling, were down-regulated in EC but up-regulated in SEs. These hormones may play different roles in cotton SE, and the exact regulatory mechanisms will be discussed in our future work.

**Fig. 5:**
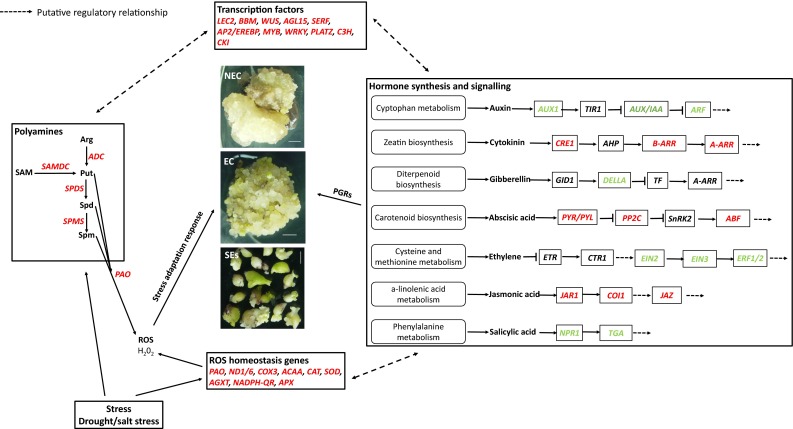
A proposed model for a role of TFs, hormones, stresses, polyamines and ROS in cotton somatic embryogenesis. *Italic words* represent genes with down- and up-regulated genes highlighted in *green* and *red* color, respectively

In conclusion, the transcriptome analysis unveiled the complex and dynamic nature of the gene network involved in cell dedifferentiation and redifferentiation during cotton SE, and identified the key regulators important for cotton SE. Our results further confirmed the main findings previously reported for cotton SE, mainly revealed the importance of genes involved in the polyamine metabolic pathways and stress response in cotton SE. Based on our results and previous reports, we proposed a working model for the potential gene network essential for successful SE (Fig. 5). In this model, we propose that TFs are the top layer players regulating reprogramming of the transcriptome required for cell dedifferentiation and redifferentiation, and that the hormone synthesis and signaling pathways, stress-response pathways and PA metabolic pathways function side-by-side and synergistically to promote embryogenic cell initiation and plantlet regeneration.

## Electronic supplementary material

Below is the link to the electronic supplementary material.

Supplementary material 1 (DOCX 7434 KB)
