# Supplementary material for: De novo transcriptome analysis reveals insights into dynamic homeostasis regulation of somatic embryogenesis in upland cotton (G. hirsutum L.)
Source: Plant Mol Biol. 2016 Aug 10;92(3):279–92. doi: 10.1007/s11103-016-0511-6 (PMC5040755; doi:10.1007/s11103-016-0511-6)
Supplement: Supplementary file 1 — Supplementary material 1 (DOCX 7434 KB) [file 11103_2016_511_MOESM1_ESM.docx]

**Supplementary Figures and tables**

**
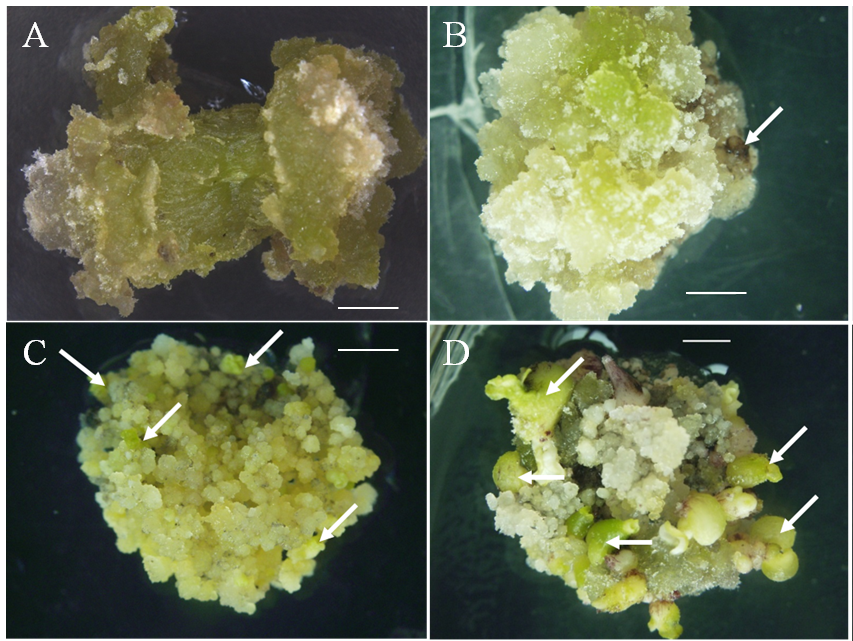
**

**Supplementary Figure 1 Samples used for RNA-sequencing.** A,NEC; B, early phase of embryogenic callus formation; C, EC; D, SEs.

**
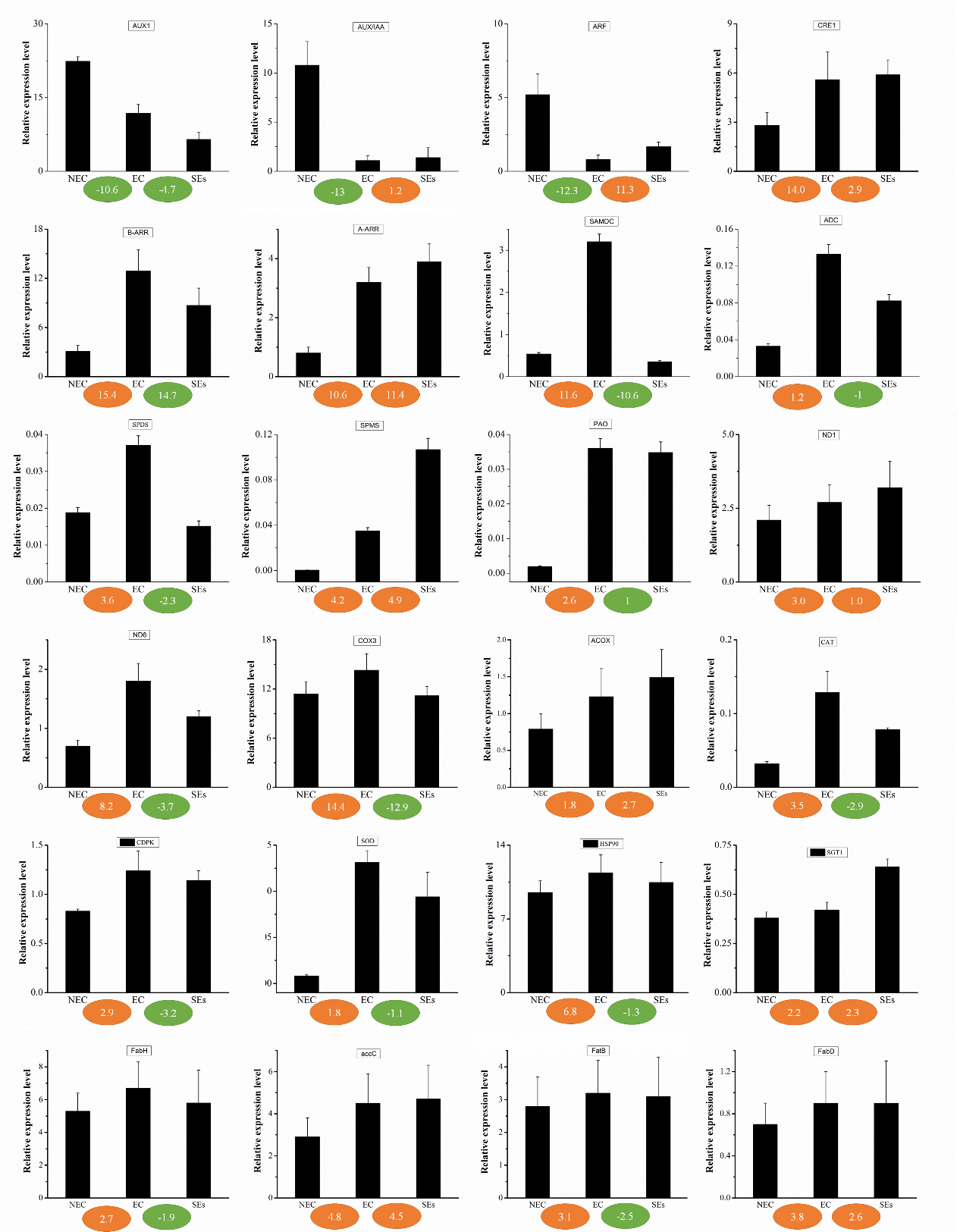
**

**Supplementary Figure 2 qRT-PCR analysis of the DEGs involved in IAA, CTK, PAs, ROS and stress response pathways.**

**
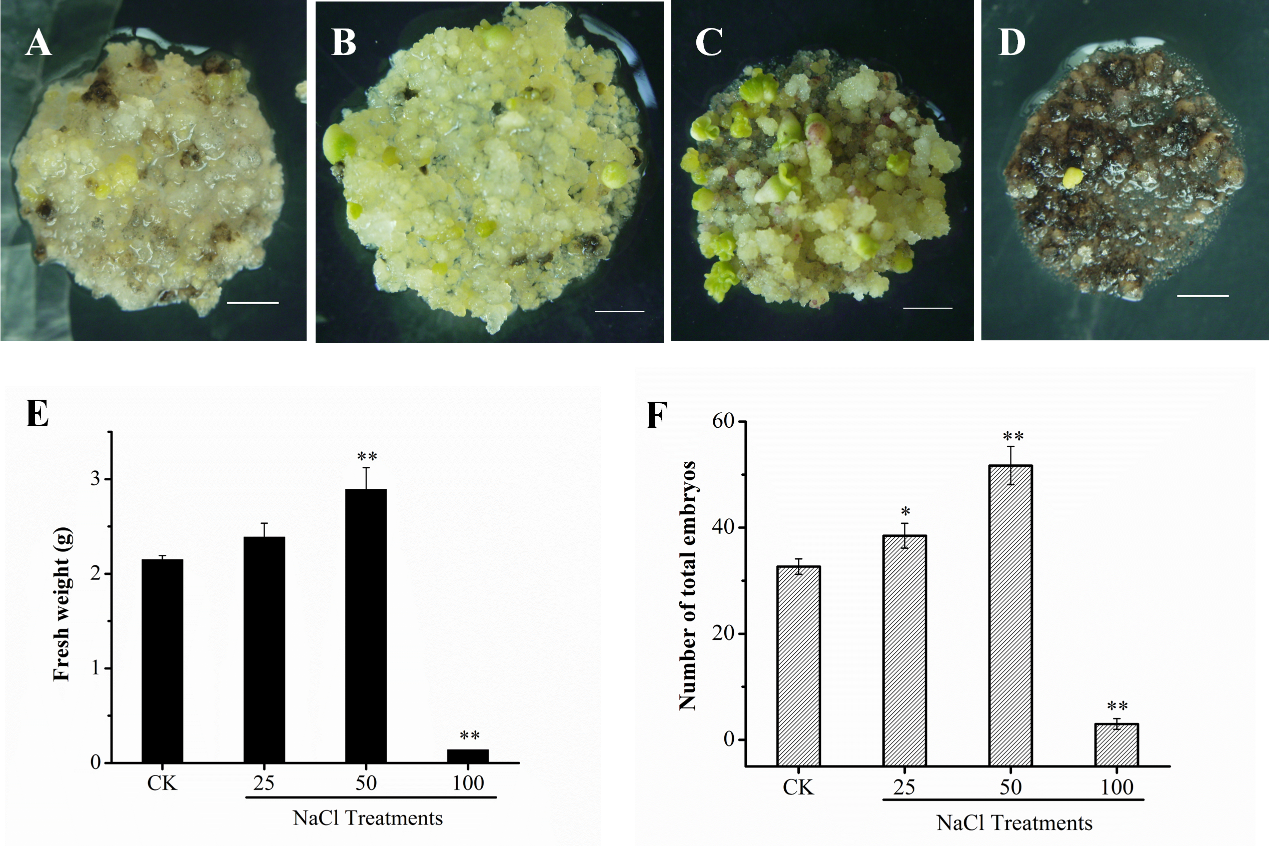
**

**Supplementary Figure 3 Effect of NaCl stress on cotton somatic embryogenesis.** A. CK (0 mM NaCl); B, 25 mM NaCl treatment; C, 50 mM NaCl treatment; D, 100 mM NaCl treatment. E, effect of NaCl on the growth of somatic embryogenic callus. F, effect of NaCl on the number of somatic embryos. * and ** indicate significant differences compared with the control (CK) at *P*<0.05 and P<0.01, respectively, according to the LSD multiple range test.

**
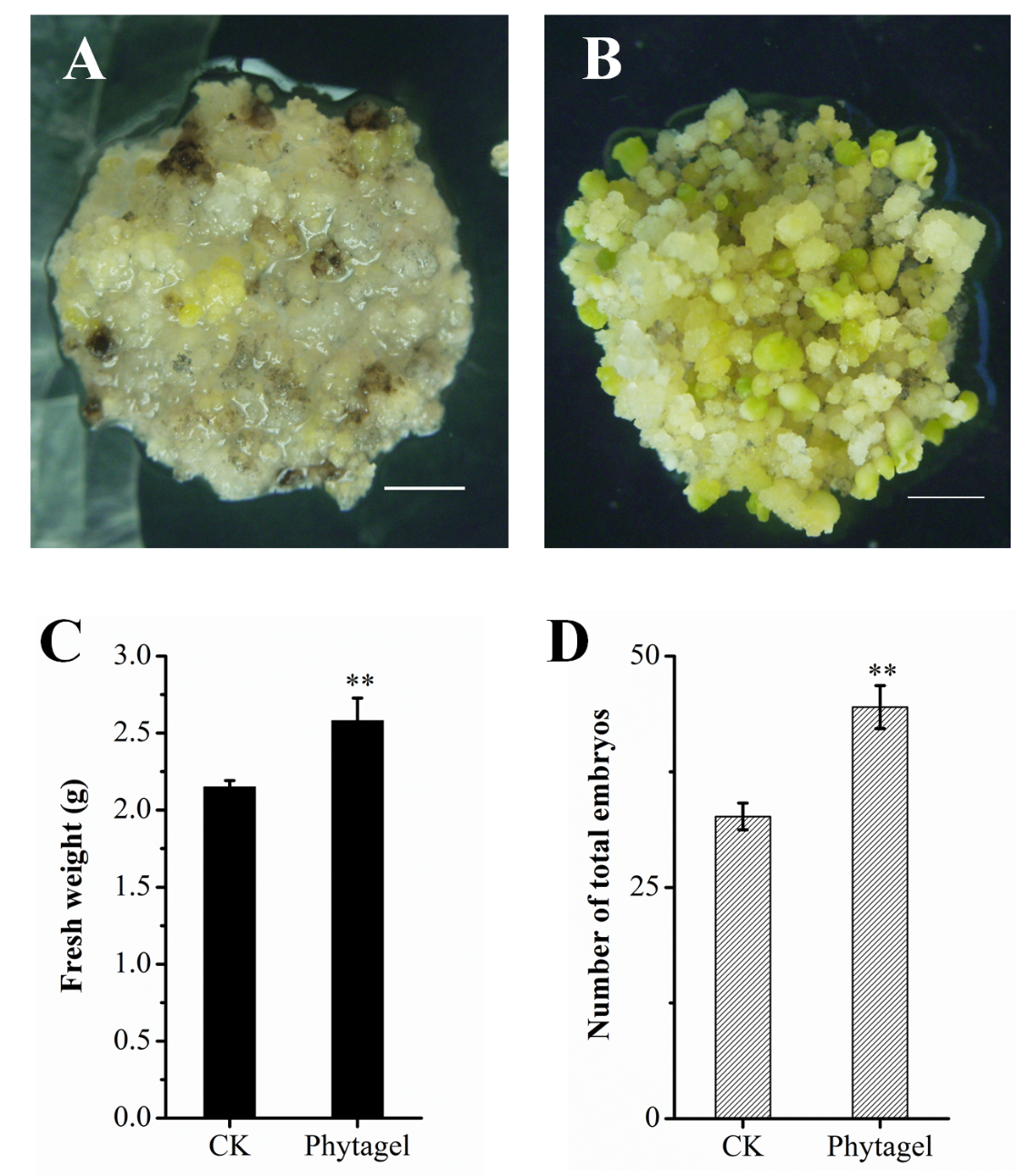
**

**Supplementary Figure 5 Effect of simulated drought stress on cotton somatic embryogenesis.** A. CK (2.5 g/L Phytagel); B, 4 g/L Phytagel treatment; C, effect of simulated drought stress on the growth of somatic embryogenic callus. D, effect of simulated drought stress on the number of somatic embryos. * and ** indicate significant differences compared with the control (CK) at *P*<0.05 and P<0.01, respectively, according to the LSD multiple range test.

**
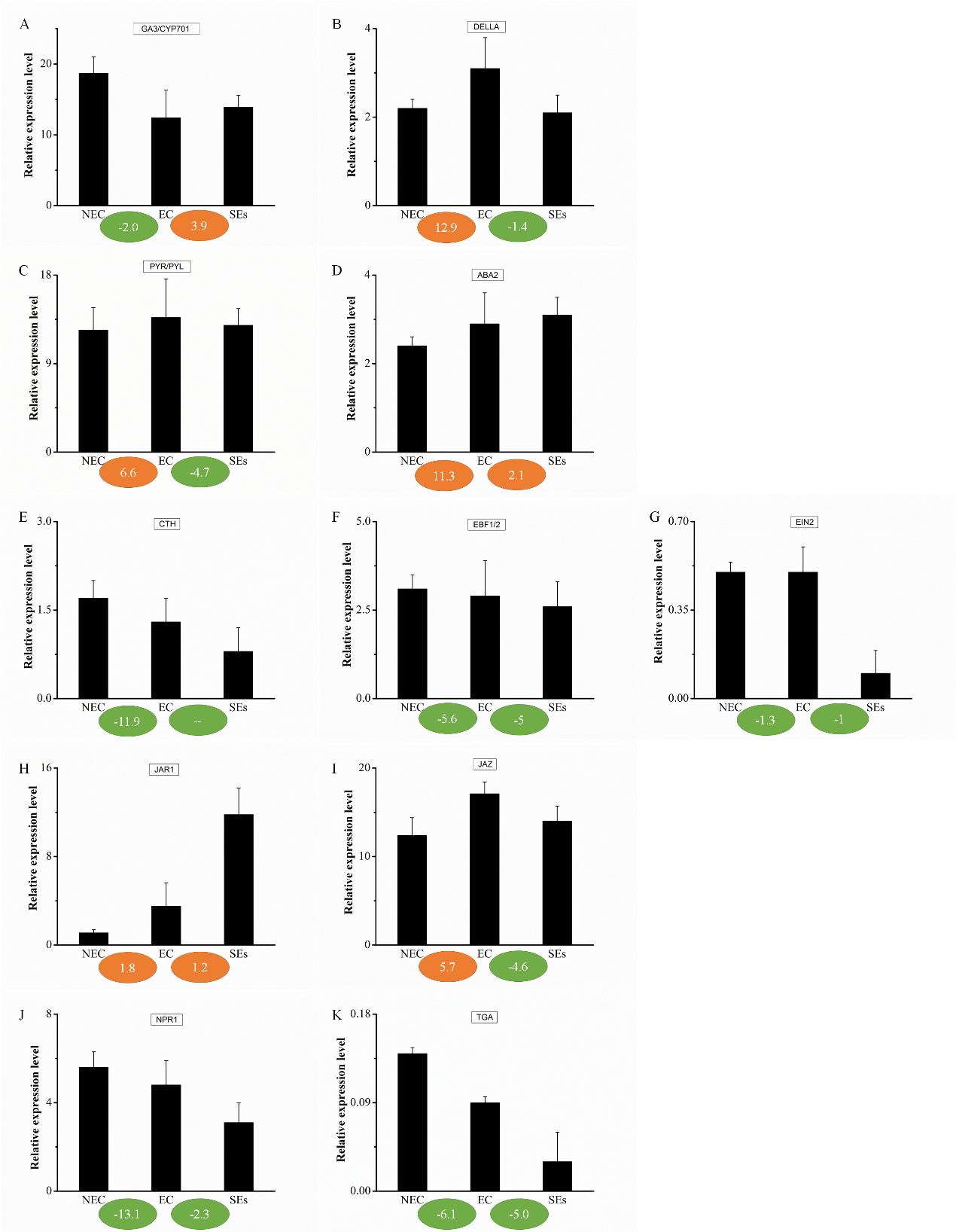
**

**Supplementary Figure 5 qRT-PCR analysis of the DEGs involved in other plant hormones.**

**Supplementary Table 1 Primers used for qRT-PCR**

| Gene ID | Gene name | Forward primer | Reverse primer |
| --- | --- | --- | --- |
| CL11109.Contig1_All | *ADC* | GAAGTCTTGCTCCGGTTGT | CTACGGCAGCAGCATCTAC |
| CL1916.Contig4_All | *SPDS* | TGAGGCTAAATGCCATTCTA | ACAAGAACCTCTTGATAGT |
| CL1916.Contig5_All | *SPMS* | ATCAGACTATCAAGAGGTC | CCAACAACCAGAACAGT |
| Unigene22850_All | *SAMDC* | GAAAACAAAAACATAAAACCATAA | GTAAAAGGATACCGTTGCG |
| Unigene16582_All | *PAO* | GAGTTACATTTAACAGCGAAGA | CTGGATAATCGCCTCAAA |
| CL2187.Contig18_All | *AUX1* | ATGCCAGGAGAGAAACAGGCAGAGG | TCGTAAGGGTAAACTC |
| CL5808.Contig3_All | *AUX/IAA* | GCAGAGATGGATAGAGATC | TCTCCTTACAGCTGACAAG |
| Unigene18856_All | *ARF* | AGAAGATAGTGACACAGATGG | TAGCATCTTCATGTACTCC |
| Unigene2420_All | *CRE1* | TCAGGAGGAACCTTGATGA | ATGGACAGCCTTATTGTA |
| Unigene17496_All | *B-ARR* | ATGGCTTCATCAAGCAATTCTC | ATTAACAGCAGCATGCAT |
| Unigene11345_All | *A-ARR* | GACATGGCGGTGGAATTAGGA | CATGCAGTAATCTGTTAT |
| Unigene19592_All | *DELLA* | ATGACTGTTACCGAGCTAG | AACGGTGACCCACTTCCT |
| Unigene27460_All | *GA3/CYP701* | AGCTATCAAATGCCTTGAAG | AAGTTGATAGGTCTTAT |
| CL7589.Contig3_All | *ABA2* | ATATGCAAGAAGAAGAGCTCG | ACAATTTTACTCTTGCA |
| CL5280.Contig2_All | *PYR/PYL* | ATGGCTTCCAGTGGGCAGCT | ACCCTTCGGCATAGTTAATA |
| Unigene217_All | *EIN2* | ATTGAATTACCAGCATCAGATT | ATGTCCGGTAAGCTATGATACT |
| Unigene2682_All | *EBF1/2* | CGGATAAGGGGTTGGTATCT | GAGGCAATCCTGTGGTT |
| Unigene38355_All | *CTH* | ACTCAGGCCATCCACGTG | AGGCCAAACTGTGCTAC |
| Unigene6360_All | *JAR1* | ATGATGGAAACTACTTTGCAGA | GAAACCAACCGAATTTCCTCAT |
| Unigene28086_All | *JAZ* | ATGTCCAGAGCTACCGTCGAGCTTGA | TCTCCAATCAACCGGATTCCG |
| Unigene32397_All | *NPR1* | AGAAGTTCTCGCCAAGCA | GATGAGTCTAATGCCCTTCTCA |
| CL3069.Contig6_All | *TGA* | ATGACAATATACGAGCAACTAAAC | TCAAACGACTTGATTCTAAT |
| CL11268.Contig2_All | *ND1* | AGTATCAAGGTGATTCTGT | AGGGAGCCATTGAAAGGT |
| CL4559.Contig2_All | *ND6* | ACGAAGATGTATTACGTCAAGA | TGGCGTGCCAGGTCACT |
| CL3850.Contig1_All | *COX3* | ACTACAGGAATAGTATTATGAT | AGTTGTATTTAATAGTGGGAT |
| CL5613.Contig1_All | *ACOX* | TAGCTGTCCTGATGCAAGC | CGGCAATATTTCGAACT |
| CL2833.Contig1_All | *CAT* | ATGTGAAATTCCATTGGAAACCCA | TCAAACCTATCCTCATGAAG |
| CL5637.Contig1_All | *APX* | AGAACTGTGCTCCACTCATGCT | GTCAGCGTATGAAAGGATA |
| CL8467.Contig1_All | *SOD* | GGAAGAGCTGTAGTTGT | GCCTTGCAGACCAATA |
| CL7474.Contig1_All | *CDPK* | ATGGGAAATGTTTGTGCTACAT | GGCTTAGGGTCTCTTCT |
| CL10919.Contig1_All | *HSP90* | ATCGGACGCTCTGGATA | TGGTGCCCGACTTGGCA |
| Unigene15666_All | *SGT1* | ACCTGGGGAAGTGGTTGTC | AACACATCATATCTGCACT |
| CL3327.Contig4_All | *FabH* | ATGGCCAAGGCATCGGGT | AGAAGTATCAACTAT |
| Unigene24187_All | *accC* | ATGCACAAGTCTGCAATG | TAGCCTCTCAGCTTCT |
| Unigene8973_All | *FatB* | ATGGTTGCCACTGCTGCTACATC | CTGACACCATCTTCGTTCT |
| Unigene26934_All | *FabD* | ACTGTCATAAGCCAGCCTGC | GTTGGCAGCATCACACAAT |
| XM_012634824 | *GhUBI* | CAGATCTTCGTCAAAACCCT | GACTCCTTCTGGATGTTGTA |

**Supplementary Table 2 Summary of Illumina RNA-sequencing results**

| **Samples** | **Total Raw Reads** | **Total Clean Reads** | **Total Clean Nucleotides (nt)** | **Q20 percentage** | **N percentage** | **GC percentage** |
| --- | --- | --- | --- | --- | --- | --- |
| **NEC** | 57,086,576 | 54,159,156 | 4,874,324,040 | 98.31% | 0.01% | 44.48% |
| **EC** | 55,006,870 | 52,737,304 | 4,746,357,360 | 98.57% | 0.01% | 43.63% |
| **SEs** | 56,102,942 | 53,511,272 | 4,816,014,480 | 98.00% | 0.02% | 44.08% |

**Supplementary Table 3** **Summary of the de novo transcript assembly**

| **Length (bp)** | **Number of Contig** | | | **Number of Unigene** | | |
| --- | --- | --- | --- | --- | --- | --- |
|  | **NEC** | **EC** | **SEs** | **NEC** | **EC** | **SEs** |
| **200-500** | 140013 | 116867 | 116341 | 60646 | 45828 | 45820 |
| **500-1000** | 13342 | 12125 | 12373 | 16278 | 16427 | 15628 |
| **1000-2000** | 8543 | 7953 | 8163 | 14348 | 16756 | 15027 |
| **2000-3000** | 2367 | 2522 | 2532 | 4664 | 6173 | 5353 |
| **>=3000** | 1016 | 1170 | 1153 | 1921 | 2813 | 2348 |

**Supplementary Table 4 TFs in NEC vs EC**

| **Gene ID** | **TF family** | **Included Domain** |
| --- | --- | --- |
| Unigene23477_All | AP2-EREBP | AP2 |
| Unigene224_All | AP2-EREBP | AP2 |
| CL4301.Contig3_All | AP2-EREBP | AP2 |
| Unigene10865_All | AP2-EREBP | AP2 |
| CL11585.Contig1_All | AP2-EREBP | AP2 |
| CL3731.Contig3_All | AP2-EREBP | AP2 |
| CL4798.Contig2_All | AP2-EREBP | AP2 |
| CL152.Contig8_All | AP2-EREBP | AP2 |
| Unigene5687_All | AP2-EREBP | AP2 |
| CL7801.Contig1_All | AP2-EREBP | AP2 |
| CL3317.Contig2_All | AP2-EREBP | AP2 |
| Unigene9217_All | AP2-EREBP | AP2 |
| CL1224.Contig1_All | AP2-EREBP | AP2 |
| Unigene13019_All | AP2-EREBP | AP2 |
| CL12231.Contig3_All | AP2-EREBP | AP2 |
| CL263.Contig4_All | AP2-EREBP | AP2 |
| CL516.Contig1_All | AP2-EREBP | AP2 |
| CL10761.Contig1_All | AP2-EREBP | AP2 |
| CL2315.Contig13_All | C2C2-CO-like | CCT,zf-B_box |
| Unigene2443_All | C2C2-Dof | zf-Dof |
| CL6732.Contig1_All | C2C2-Dof | zf-Dof |
| Unigene1151_All | C2C2-GATA | GATA |
| Unigene48165_All | C2C2-GATA | GATA |
| CL366.Contig5_All | C2C2-GATA | GATA |
| Unigene5878_All | C2C2-GATA | GATA |
| Unigene39480_All | C2C2-GATA | GATA |
| Unigene21349_All | C2C2-GATA | GATA |
| CL3586.Contig1_All | C2C2-GATA | GATA |
| Unigene49909_All | C2H2 | zf-C2H2 |
| CL7401.Contig2_All | C2H2 | zf-C2H2 |
| Unigene9035_All | C2H2 | zf-C2H2 |
| CL4689.Contig2_All | C2H2 | zf-C2H2 |
| Unigene30528_All | C2H2 | zf-C2H2 |
| Unigene39038_All | C2H2 | zf-C2H2 |
| Unigene40035_All | C2H2 | zf-C2H2 |
| Unigene39590_All | C2H2 | zf-C2H2 |
| CL3617.Contig1_All | C3H | zf-CCCH |
| Unigene18500_All | C3H | zf-CCCH |
| CL1707.Contig1_All | C3H | zf-CCCH |
| CL5804.Contig1_All | C3H | zf-CCCH |
| CL755.Contig5_All | C3H | zf-CCCH |
| CL5782.Contig1_All | C3H | zf-CCCH |
| Unigene18235_All | C3H | zf-CCCH |
| CL11277.Contig2_All | C3H | zf-CCCH |
| CL1707.Contig4_All | C3H | zf-CCCH |
| CL11341.Contig1_All | C3H | zf-CCCH |
| CL2342.Contig2_All | C3H | zf-CCCH |
| CL755.Contig2_All | C3H | zf-CCCH |
| Unigene27493_All | C3H | zf-CCCH |
| CL7870.Contig1_All | C3H | zf-CCCH |
| CL1328.Contig3_All | C3H | zf-CCCH |
| CL10465.Contig2_All | C3H | zf-CCCH |
| CL1193.Contig8_All | C3H | zf-CCCH |
| CL5804.Contig6_All | C3H | zf-CCCH |
| Unigene34230_All | C3H | zf-CCCH |
| Unigene41020_All | C3H | zf-CCCH |
| CL6765.Contig1_All | C3H | zf-CCCH |
| CL584.Contig5_All | C3H | zf-CCCH |
| CL1925.Contig1_All | C3H | zf-CCCH |
| CL7325.Contig1_All | MYB | Myb_DNA-binding |
| Unigene5684_All | MYB | Myb_DNA-binding |
| CL8154.Contig4_All | MYB | Myb_DNA-binding |
| CL8154.Contig5_All | MYB | Myb_DNA-binding |
| Unigene6685_All | MYB | Myb_DNA-binding |
| Unigene21163_All | MYB | Myb_DNA-binding |
| Unigene2484_All | MYB | Myb_DNA-binding |
| CL5849.Contig2_All | MYB | Myb_DNA-binding |
| Unigene50713_All | MYB | Myb_DNA-binding |
| CL11462.Contig1_All | MYB | Myb_DNA-binding |
| CL7700.Contig4_All | MYB | Myb_DNA-binding |
| CL325.Contig8_All | MYB | Myb_DNA-binding |
| CL2061.Contig3_All | MYB | Myb_DNA-binding |
| CL6623.Contig1_All | MYB | Myb_DNA-binding |
| CL8097.Contig7_All | MYB | Myb_DNA-binding |
| CL4984.Contig4_All | MYB | Myb_DNA-binding |
| CL1337.Contig9_All | MYB | Myb_DNA-binding |
| CL4984.Contig2_All | MYB | Myb_DNA-binding |
| Unigene3277_All | MYB | Myb_DNA-binding |
| CL4139.Contig1_All | MYB | Myb_DNA-binding |
| Unigene364_All | MYB | Myb_DNA-binding |
| CL11976.Contig1_All | MYB | Myb_DNA-binding |
| Unigene9249_All | MYB | Myb_DNA-binding |
| CL4139.Contig5_All | MYB | Myb_DNA-binding |
| CL11498.Contig1_All | MYB | Myb_DNA-binding |
| Unigene35292_All | MYB | Myb_DNA-binding |
| CL3550.Contig3_All | MYB | Myb_DNA-binding |
| Unigene29101_All | MYB | Myb_DNA-binding |
| Unigene2577_All | MYB | Myb_DNA-binding |
| CL6857.Contig1_All | MYB | Myb_DNA-binding |
| CL1039.Contig3_All | MYB | Myb_DNA-binding |
| CL11916.Contig1_All | MYB | Myb_DNA-binding |
| Unigene31150_All | MYB | Myb_DNA-binding |
| CL1703.Contig8_All | MYB | Myb_DNA-binding |
| Unigene23741_All | MYB | Myb_DNA-binding |
| Unigene23955_All | MYB | Myb_DNA-binding |
| Unigene23744_All | MYB | Myb_DNA-binding |
| Unigene24186_All | MYB | Myb_DNA-binding |
| Unigene23742_All | MYB | Myb_DNA-binding |
| Unigene10866_All | MYB | Myb_DNA-binding |
| CL7325.Contig1_All | MYB-related | Myb_DNA-binding |
| Unigene5684_All | MYB-related | Myb_DNA-binding |
| Unigene6685_All | MYB-related | Myb_DNA-binding |
| Unigene21163_All | MYB-related | Myb_DNA-binding |
| Unigene2484_All | MYB-related | Myb_DNA-binding |
| CL5849.Contig2_All | MYB-related | Myb_DNA-binding |
| Unigene50713_All | MYB-related | Myb_DNA-binding |
| CL11462.Contig1_All | MYB-related | Myb_DNA-binding |
| CL325.Contig8_All | MYB-related | Myb_DNA-binding |
| CL2061.Contig3_All | MYB-related | Myb_DNA-binding |
| CL6623.Contig1_All | MYB-related | Myb_DNA-binding |
| CL8097.Contig7_All | MYB-related | Myb_DNA-binding |
| CL4984.Contig4_All | MYB-related | Myb_DNA-binding |
| CL4984.Contig2_All | MYB-related | Myb_DNA-binding |
| Unigene3277_All | MYB-related | Myb_DNA-binding |
| CL4139.Contig1_All | MYB-related | Myb_DNA-binding |
| Unigene364_All | MYB-related | Myb_DNA-binding |
| CL11976.Contig1_All | MYB-related | Myb_DNA-binding |
| Unigene9249_All | MYB-related | Myb_DNA-binding |
| CL4139.Contig5_All | MYB-related | Myb_DNA-binding |
| CL11498.Contig1_All | MYB-related | Myb_DNA-binding |
| Unigene35292_All | MYB-related | Myb_DNA-binding |
| CL3550.Contig3_All | MYB-related | Myb_DNA-binding |
| Unigene29101_All | MYB-related | Myb_DNA-binding |
| Unigene2577_All | MYB-related | Myb_DNA-binding |
| CL6857.Contig1_All | MYB-related | Myb_DNA-binding |
| CL1039.Contig3_All | MYB-related | Myb_DNA-binding |
| CL11916.Contig1_All | MYB-related | Myb_DNA-binding |
| Unigene31150_All | MYB-related | Myb_DNA-binding |
| CL1703.Contig8_All | MYB-related | Myb_DNA-binding |
| Unigene23741_All | MYB-related | Myb_DNA-binding |
| Unigene23955_All | MYB-related | Myb_DNA-binding |
| Unigene23744_All | MYB-related | Myb_DNA-binding |
| Unigene24186_All | MYB-related | Myb_DNA-binding |
| Unigene23742_All | MYB-related | Myb_DNA-binding |
| Unigene10866_All | MYB-related | Myb_DNA-binding |
| Unigene20325_All | WRKY | WRKY |
| CL8394.Contig1_All | WRKY | WRKY |
| CL12269.Contig2_All | WRKY | WRKY |
| CL8394.Contig3_All | WRKY | WRKY |
| CL11363.Contig1_All | WRKY | WRKY |
| Unigene9852_All | WRKY | WRKY |
| CL6783.Contig1_All | WRKY | WRKY |
| Unigene54572_All | WRKY | WRKY |
| CL12073.Contig2_All | WRKY | WRKY |
| CL7343.Contig4_All | WRKY | WRKY |

**Supplementary Table 4 TFs in EC vs SEs**

| **Gene ID** | **TF family** | **Included Domain** |
| --- | --- | --- |
| CL10761.Contig1_All | AP2-EREBP | AP2 |
| CL3317.Contig2_All | AP2-EREBP | AP2 |
| CL7801.Contig1_All | AP2-EREBP | AP2 |
| Unigene21225_All | AP2-EREBP | AP2 |
| Unigene21713_All | AP2-EREBP | AP2 |
| Unigene21715_All | AP2-EREBP | AP2 |
| Unigene224_All | AP2-EREBP | AP2 |
| Unigene28663_All | AP2-EREBP | AP2 |
| Unigene9815_All | AP2-EREBP | AP2 |
| CL3547.Contig3_All | C2C2-GATA | GATA |
| Unigene21349_All | C2C2-GATA | GATA |
| Unigene48165_All | C2C2-GATA | GATA |
| Unigene5878_All | C2C2-GATA | GATA |
| CL679.Contig2_All | C2H2 | zf-C2H2 |
| Unigene49909_All | C2H2 | zf-C2H2 |
| CL3617.Contig1_All | C3H | zf-CCCH |
| CL6765.Contig1_All | C3H | zf-CCCH |
| Unigene18500_All | C3H | zf-CCCH |
| CL11462.Contig1_All | MYB | Myb_DNA-binding |
| CL11498.Contig1_All | MYB | Myb_DNA-binding |
| CL11916.Contig1_All | MYB | Myb_DNA-binding |
| CL3550.Contig3_All | MYB | Myb_DNA-binding |
| CL6857.Contig1_All | MYB | Myb_DNA-binding |
| CL7325.Contig1_All | MYB | Myb_DNA-binding |
| Unigene10866_All | MYB | Myb_DNA-binding |
| Unigene21163_All | MYB | Myb_DNA-binding |
| Unigene24186_All | MYB | Myb_DNA-binding |
| Unigene2577_All | MYB | Myb_DNA-binding |
| Unigene29101_All | MYB | Myb_DNA-binding |
| Unigene50713_All | MYB | Myb_DNA-binding |
| Unigene6685_All | MYB | Myb_DNA-binding |
| CL11462.Contig1_All | MYB-related | Myb_DNA-binding |
| CL11498.Contig1_All | MYB-related | Myb_DNA-binding |
| CL11916.Contig1_All | MYB-related | Myb_DNA-binding |
| CL3550.Contig3_All | MYB-related | Myb_DNA-binding |
| CL6857.Contig1_All | MYB-related | Myb_DNA-binding |
| CL7325.Contig1_All | MYB-related | Myb_DNA-binding |
| Unigene10866_All | MYB-related | Myb_DNA-binding |
| Unigene21163_All | MYB-related | Myb_DNA-binding |
| Unigene24186_All | MYB-related | Myb_DNA-binding |
| Unigene2577_All | MYB-related | Myb_DNA-binding |
| Unigene29101_All | MYB-related | Myb_DNA-binding |
| Unigene50713_All | MYB-related | Myb_DNA-binding |
| Unigene6685_All | MYB-related | Myb_DNA-binding |
| CL6783.Contig1_All | WRKY | WRKY |
| CL7866.Contig1_All | WRKY | WRKY |
| CL8394.Contig2_All | WRKY | WRKY |
| CL8394.Contig3_All | WRKY | WRKY |
| Unigene54572_All | WRKY | WRKY |
| Unigene55406_All | WRKY | WRKY |
| Unigene9852_All | WRKY | WRKY |
